# Prevalence and Clinical Impact of Restless Legs Syndrome in Pediatric Populations with Attention-Deficit/Hyperactivity Disorder: A Systematic Review

**DOI:** 10.3390/clockssleep7030050

**Published:** 2025-09-17

**Authors:** Toni Ghayad, Anaïs Mungo, Matthieu Hein

**Affiliations:** 1 Faculté de Médecine, Université Libre de Bruxelles (ULB), 1070 Bruxelles, Belgium; toni.ghayad@ulb.be; 2 Centre Hospitalier Le Domaine-ULB, Service de Psychiatrie, Université Libre de Bruxelles (ULB), 1420 Braine l’Alleud, Belgium; anais.mungo@ulb.be; 3 CHU Brugmann, Service de Psychiatrie et Laboratoire du Sommeil, Université libre de Bruxelles (ULB), Place A. Van Gehuchten, 4, 1020 Bruxelles, Belgium; 4 Laboratoire de Psychologie Médicale et Addictologie (ULB312), Université Libre de Bruxelles (ULB), Place A. Van Gehuchten, 4, 1020 Bruxelles, Belgium

**Keywords:** Attention Deficit Hyperactivity Disorder, restless legs syndrome, prevalence, pediatric population

## Abstract

Attention Deficit Hyperactivity Disorder (ADHD) is a prevalent disorder in the pediatric population. Furthermore, there appears to be a special relationship between ADHD and Restless Legs Syndrome (RLS). The objective of this review was therefore to provide an updated overview of the current literature regarding the prevalence of RLS and its potential clinical impact in pediatric ADHD subjects (<18 years). A systematic literature review was carried out in May 2025 in the PubMed-Medline database according to PRISMA criteria. After evaluation by two readers of the 147 identified articles, 9 articles investigating the prevalence of RLS with or without assessment of its potential clinical impact were selected for this systematic literature review. The prevalence of RLS in children and adolescents with ADHD showed significant variation, ranging from 11% to 54%. One study found a significant impact of RLS on academic performance and life skills in pediatric ADHD subjects. Three studies highlighted higher severity of ADHD complaints in subjects with comorbid RLS. One study reported higher RLS severity scores in the ADHD+RLS group and significantly more severe scores in the “hyperactive-impulsive” ADHD subtype. Two studies identified a significant association between a family history of RLS and RLS+ADHD comorbidity. Compared with the general pediatric population, the prevalence of RLS appears to be higher in pediatric ADHD subjects. Finally, this comorbid sleep disorder could worsen the severity of ADHD symptoms and complicate its clinical management.

## 1. Introduction

Attention Deficit Hyperactivity Disorder (ADHD) is a disorder characterized by symptoms of hyperactivity, inattention, and/or impulsivity, with cognitive, behavioral, emotional, social, and/or academic impairments [1]. ADHD may be diagnosed as early as 4 years of age, with symptoms appearing before the age of 12 [1]. The symptoms are divided into two main categories: hyperactivity/impulsivity and inattention. According to the DSM-5-TR, the diagnosis of ADHD also requires impairment in at least two areas of the person’s functioning: academic, social, or occupational [1].

The pathophysiology of ADHD remains incompletely understood [2,3,4]. Current evidence suggests that it involves an imbalance in catecholamine metabolism within the cerebral cortex [2,3,4]. Importantly, dopamine (DA) and noradrenaline (NA) originate from distinct anatomical substrates: NA is predominantly synthesized by neurons of the locus coeruleus, whereas DA is mainly produced in the substantia nigra, as well as by dopaminergic cell groups in the thalamus and basal forebrain. This neurochemical imbalance is supported by findings from animal models, functional brain imaging studies, and the clinical efficacy of pharmacological treatments—particularly noradrenergic agents—that enhance extracellular dopamine levels in the brain [2,3,4]. In addition, environmental and epigenetic factors, such as iron deficiency, are thought to contribute secondarily, although their overall significance remains a matter of debate [2,3,4]. Among the other described environmental factors are prenatal factors, such as smoking, alcohol, or substance use during pregnancy [2,3,4]. Maternal stress and hypothyroidism are also mentioned [2,3,4]. Social factors, such as social deprivation, have also been linked to the development of ADHD [2,3,4]. Additionally, a clear genetic link has been established in several studies [5].

ADHD is a common disorder, with an estimated prevalence of around 5.29% worldwide, 5% in Europe, but ranging from 5 to 18% in the United States [6]. This disorder is also associated with many comorbidities, including oppositional defiant disorder, conduct disorder, depression, anxiety disorders, learning disorders, and sleep disorders (including restless legs syndrome [RLS]) [7].

Regarding the management of ADHD, a holistic approach to the management of the disorder should be implemented, including parent and teacher psychoeducation [8,9]. For children aged between 4 and 5 years, behavioral therapies are recommended over pharmacological treatment, and pharmacological treatment should only be considered if behavioral therapy fails to improve the child’s functioning, with a preference for methylphenidate over amphetamines and non-stimulant treatments [8,9]. For children over 6 years of age and adolescents, first-line treatment includes stimulant medication combined with behavioral therapy [8,9]. Treatment response is assessed clinically and through scales such as the Conners Parent and Teacher Rating Scales, the ADHD Rating Scale, the Vanderbilt Rating Scale, and the Swanson, Nolan, and Pelham Rating Scale (SNAP-IV) [8,9].

The management of comorbid sleep disorders for ADHD subjects is essential due to their bidirectional influence on each other and the suboptimal treatment response due to a comorbidity [10]. Regarding sleep disorders, one study evaluated the effect of a brief sleep hygiene and behavior intervention in two sessions in 244 randomized children with ADHD and at least one sleep disorder, with modest improvement in ADHD symptoms at 3- and 6-month follow-ups, highlighting the importance of managing comorbidities in ADHD, particularly sleep disorders [11]. Within this context, restless legs syndrome (RLS) is of particular interest, as its association with ADHD may, at least in part, be mediated through the sleep disturbances it produces in pediatric patients. By causing chronic sleep deprivation due to difficulties initiating and maintaining sleep, RLS may exacerbate or even mimic ADHD symptoms such as inattention, hyperactivity, and impulsivity. Several conceptual frameworks have been proposed to explain this association: ADHD may inherently cause sleep problems; sleep disorders such as RLS may trigger or mimic ADHD symptoms; ADHD and RLS may interact through reciprocal causation; or both conditions may share a common neurobiological origin [11]. Clarifying the temporal sequence of symptom onset is therefore critical, as it is plausible that RLS may occur first, with the resulting sleep deprivation contributing—at least in some cases—to the emergence of ADHD symptoms. This hypothesis reinforces the need for systematic assessment of sleep disorders in ADHD patients. Moreover, effectively addressing RLS-related sleep disruption could be a key component in improving ADHD outcomes, as better sleep quality has the potential to reduce both sleep-specific and ADHD-related symptoms [11].

RLS or Willis-Ekbom Syndrome is a neurological disorder initially described by Willis in 1685 and later defined by Ekbom in 1944. Pediatric RLS was described only in 1994 with diagnostic criteria introduced in 2003 and last updated in 2013 [12]. Despite this, RLS remains overlooked and neglected in clinical practice, especially in the pediatric population, where complaints mainly revolve around insomnia or oppositional behavior rather than the classic symptoms of RLS [13]. Studies have also demonstrated that the presence of RLS increases the risk of psychiatric comorbidities, notably ADHD [14]. This observation appears to be due to a bidirectional relationship between RLS and psychiatric comorbidities as well as the side effects of administered psychotropic treatments and the pharmacosusceptibility of some patients [14].

RLS is primarily a clinical diagnosis, based on established international criteria, and does not require polysomnography for confirmation. However, polysomnography may be useful in selected cases to assess the consequences of RLS on sleep architecture and continuity, including the detection of periodic limb movements, quantification of sleep fragmentation, and evaluation of total sleep time, thereby providing additional insights into its potential impact on daytime functioning and ADHD symptoms. RLS is quite common in children and adolescents, with an estimated prevalence of 2–4% [15]. However, it is often underestimated in clinical practice due to various reasons, including trivialization by healthcare practitioners, misdiagnosis, and the inability of young children to verbalize their complaints [16]. Thus, the 3rd International Classification of Sleep Disorders (ICSD-3) suggests that for diagnosing RLS in a child, the symptoms description should be made using the subject’s own words [17].

Family history is also fundamental. It is found in 40–92% of pediatric RLS cases, and most cases with early onset (before 35 years), are familial [18]. It is also essential to assess daytime complaints, including cognitive impairments and academic decline, and mood alterations such as mood swings, irritability, or sadness [19,20]. Special attention should be given to iatrogenic RLS, secondary to medication treatments, especially those used in pediatrics, such as fluoxetine and escitalopram. The choice of antidepressants in the pediatric population should consider the potential induction or exacerbation of RLS [19,20].

Among the psychiatric comorbidities of RLS in the pediatric population, the most well-known associations are with ADHD, oppositional defiant disorder (ODD), anxiety disorders, and depression, with the link between ADHD and RLS being the most well-described [21,22]. On the other hand, the least investigated link in the literature seems to be that of RLS and depressive disorder in children [21,22]. It should also be noted that there is no clear distinction between adolescents and prepubescent subjects in most studies investigating the links between RLS and psychiatric disorders in this population [21,22].

The pathophysiology of RLS remains complex and not fully elucidated, with numerous theories currently proposed, especially related to the central nervous system. The main mechanisms involve depletion of central iron stores, as well as alterations in dopaminergic systems, circadian rhythm physiology, thalamic function, and other neurotransmitter disturbances, such as glutamate and gamma-aminobutyric acid (GABA) [23,24,25].

A deficiency in central iron reserves, despite normal blood iron levels, is a relatively common finding in RLS, demonstrated by lower cerebrospinal fluid ferritin levels in subjects with RLS, as well as lower iron reserves in the red, thalamic, and striatal nuclei [26]. An autopsy study revealed a decrease in transferrin receptors, suggesting a more complex pathophysiological process than simple iron deficiency (involving a defect in the ferric regulatory protein 1 in neuromelanin-containing cells) [27]. The theory of dopaminergic depletion is also advanced [28]. Many studies also show thalamic abnormalities in patients with RLS, including anatomical, metabolic, and functional aspects [29].

Regarding the management of RLS, it primarily relies on two types of measures: non-pharmacological and pharmacological [30,31]. Non-pharmacological measures include strict sleep hygiene, avoidance of psychostimulants, moderate physical activity, or walking a few hours before bedtime [30,31]. For pharmacological measures, iron supplementation remains the first-line treatment if a deficiency is identified, with possible adjustments of treatments that may induce iatrogenic RLS [30,31]. There are currently no treatments with approval from European scientific societies or the Food and Drug Administration (FDA) for the treatment of RLS in the pediatric population [30,31]. Therefore, the rationale for proposed treatments is based on empirical data from adults, case series, and case reports in pediatrics [29,30]. Some trials have been conducted with Clonazepam, a long-acting benzodiazepine, and Pramipexole, a dopaminergic agonist [32]. Clonidine, an alpha-2 adrenergic agonist, is also combined with iron and may improve sleep onset in children [33].

In conclusion, RLS is a syndrome with a significant prevalence in the pediatric population and is strongly associated with ADHD. In this context, the main objective of this systematic literature review was to investigate the prevalence of RLS in pediatric ADHD subjects (<18 years) in order to have reliable data concerning the frequency of this comorbidity in this particular subpopulation. Finally, from the articles selected for this prevalence assessment, the secondary objective of this systematic literature review was to assess the potential impact of RLS on the clinical presentation and therapeutic management of ADHD in the pediatric population.

## 2. Methods

### 2.1. Article Selection

Based on PRISMA (Preferred Reporting Items for Systematic Reviews and Meta-Analyses) criteria, a systematic review of the literature investigating the prevalence of RLS with or without assessment of its potential clinical impact in pediatric ADHD subjects (<18 years) was performed between 1 May 2025 and 31 May 2025 in the PubMed-Medline database with the keyword sequence: (Attention Deficit Disorder with Hyperactivity [MeSH Terms] OR Attention Deficit Disorder with Hyperactivity) AND (Restless Legs Syndrome [MeSH Terms] OR Restless Legs Syndrome). The review protocol was registered in PROSPERO (CRD420251123050) and conducted in accordance with the PRISMA 2020 guidelines. Furthermore, this protocol has not been previously published.

After using this sequence of keywords, 147 articles were identified and evaluated by two readers according to the following inclusion criteria:

- Articles investigating the prevalence of RLS with or without assessment of its potential clinical impact in pediatric ADHD subjects (<18 years).
- ADHD diagnosed according to DSM-IV or DSM 5 diagnostic criteria.
- RLS diagnosed during clinical interview using standardized diagnostic criteria (International Classification of Sleep Disorders or International Restless Legs Syndrome Study Group) or using questionnaires validated for clinical research.
- Any study design (cross-sectional, longitudinal, prospective, retrospective, interventional and experimental) except for literature reviews case reports and letters to editor.
- Articles written in English or French.
- Articles available in full version.

**Figure 1:**
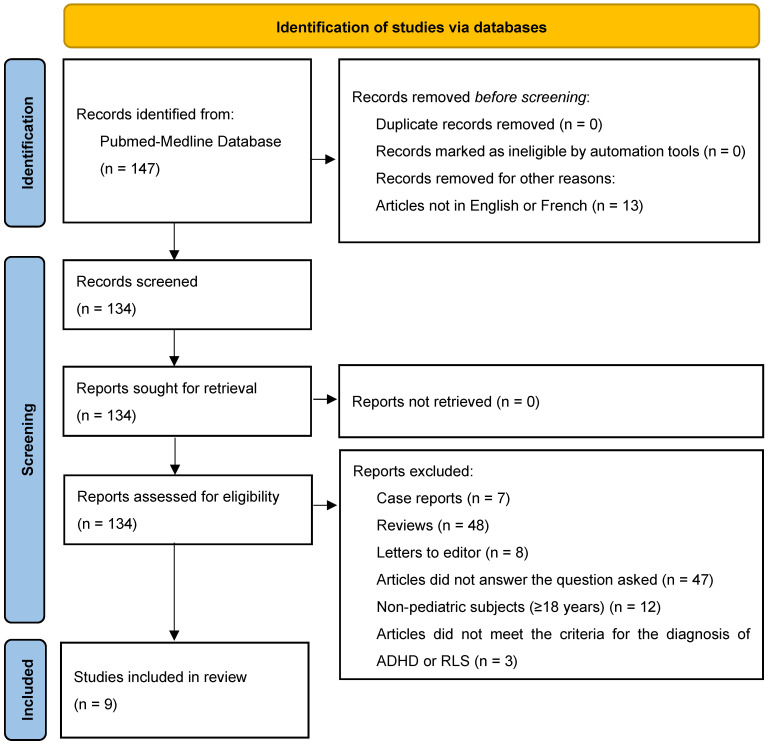
Article selection diagram.

After evaluation based on these criteria, 9 articles investigating the prevalence of RLS with or without assessment of its potential clinical impact in pediatric ADHD subjects were finally selected for inclusion in this systematic literature review (Figure 1).

### 2.2. Quality Assessment of Articles

The quality of the studies included in this systematic literature review was evaluated using the French recommendations of the National Agency for Accreditation and Evaluation in Health (integrated with the *Haute Autorité de Santé*) [34]. Based on these recommendations, 3 grades of recommendations are determined based on the level of scientific evidence:

- Grade A (established scientific evidence) for level 1 of scientific evidence (high-powered randomized comparative trial, meta-analysis of randomized comparative trials, and decision analysis based on well-conducted studies);
- Grade B (presumed scientific evidence) for level 2 of scientific evidence (low-powered randomized comparative trials, well-conducted non-randomized comparative studies, and cohort studies);
- Grade C (low level of scientific evidence) for level 3 of scientific evidence (case–control studies) or level 4 of scientific evidence (comparative studies with significant biases, retrospective studies, case series, and descriptive epidemiological studies).

Furthermore, the risk of bias for each of the included studies was investigated using the ROBINS-I tool (Risk Of Bias In Nonrandomized Studies of Interventions) that allows an evaluation across 7 domains: bias due to confounding, bias due to selection of participants, bias in classification of interventions, bias due to deviations from intended interventions, bias due to missing data, bias in measurement of outcomes and bias in selection of the reported results [35].

### 2.3. Data Extraction

The data extracted from the 9 studies selected for the analysis of the available literature are as follows: main author, year of publication, country of the study, study design, sample size, age of the sample, criteria used for diagnosing ADHD and RLS, presence or absence of polysomnography, prevalence of RLS in the sample, other relevant evaluated parameters and main results.

## 3. Results

### 3.1. Prevalence of RLS in Pediatric ADHD Subjects

In the 9 selected articles investigating the prevalence of RLS in pediatric ADHD subjects (aged 3 to 18 years), there is a marked disparity in the different prevalence rates, ranging from 11% in Srifuengfung et al. (2020) to 54% in Konofal et al. (2007) [36,37]. Sierra Montoya et al. (2018) estimated the prevalence of RLS at 13.5% [38]. Chervin et al. (1997) found a prevalence of 15% (but with a non-significant difference compared to the group with a psychiatric disorder other than ADHD or the control group) [39]. Furthermore, the prevalence of RLS in pediatric ADHD subjects was, respectively, 19.5%, 24%, 25.4%, 26% and 33% in the studies of Kapoor et al. (2021), Chervin et al. (2002), Silvestri et al. (2009), Silvestri et al. (2007) and Oner et al. (2007) [40,41,42,43,44]. Finally, Sierra Montoya et al. (2018) is the only one among the selected studies that stratified the sample into subgroups based on age, with RLS prevalence rates among ADHD subjects of 21.8% for ages 4–8, 51.3% for ages 9–13, and 25% for ages over 13 [38]. The different prevalence rates are summarized in Table 1.

### 3.2. Clinical Impact of RLS in Pediatric ADHD Subjects

Regarding the impact on behavior, individual functioning, and the severity of ADHD (evaluated using the Conners’ Teacher Rating Scale and Parent Rating Scale, CTRS and CPRS, respectively) of the comorbidity of ADHD and RLS, Srifuengfung et al. (2020) found, after multivariate regression analysis, an effect on school functioning and life skills [36]. Oner et al. (2007) did not find a significant difference in behavioral tests [43]. Konofal et al. (2007) observed a trend towards an increased CPRS in the ADHD+RLS group compared to the ADHD group, as well as an increased hyperactivity-impulsivity index in the ADHD group compared to the ADHD+RLS group [37]. Silvestri et al. (2009) found a significant increase in hyperactivity and opposition scores (CTRS, CPRS, and SNAP-IV) in the ADHD+RLS group compared to the ADHD group, with an association of RLS with the hyperactive subtype of ADHD [42]. Finally, Silvestri et al. (2007) highlighted that RLS was associated with higher scores of inattention, impulsivity/hyperactivity and oppositional behaviors in ADHD subjects [44].

Regarding the impact on the severity of RLS in the comorbidity of ADHD and RLS (evaluated by the International Restless Leg Syndrome-Rating Scale [IRLS-RS]), Silvestri et al. (2009) found a significantly more severe IRLS-RS in the ADHD+RLS group compared to the ADHD group and significantly more severe scores in the hyperactive-impulsive or combined subtypes than in the inattentive subtype [42]. Chervin et al. (1997) evaluated RLS severity using a non-validated questionnaire submitted to parents and did not find a significant difference between the different groups [39].

Regarding the link between family history of RLS and the comorbidity of ADHD and RLS, Konofal et al. (2007) found family history of RLS in 58% of subjects in the ADHD+RLS group compared to 10% in the ADHD group but statistical analysis was not applicable [37]. Srifuengfung et al. (2020) found, after binary regression analysis, a significant association between the presence of a first-degree biological parent with RLS symptoms and the comorbidity of ADHD and RLS [36].

The potential clinical impact of RLS in pediatric ADHD subjects is summarized in Table 1.

### 3.3. Risk of Bias of Selected Studies

Overall, the included studies presented a moderate to severe risk of bias, particularly due to confounding factors, selection bias, and limitations in outcome measurement. None of the studies were randomized, and most had small sample sizes and observational designs, further limiting the robustness of the findings. The risk of bias assessment for each of the selected studies according to the ROBINS-I tool is available in Table 2.

## 4. Discussion

The aim of this systematic review was to evaluate the prevalence in the literature of RLS in pediatric ADHD subjects and to assess the potential clinical impact of this comorbidity.

The prevalences of RLS in pediatric ADHD subjects found in the literature ranged from 11% to 54% among the different included studies [36,37,38,39,40,41,42,43,44]. These prevalences exceed by far those estimated in the general pediatric population (2%) [45]. It is worth noting that Picchietti et al. (2007) evaluated the prevalence of RLS within 10,523 families in the United States and found no significant difference between the prevalence in the 8–11 years age group (1.9%) and the 12–17 years age group (2%) [15]. However, there is significant heterogeneity among the different studies included in this systematic review for the prevalence rates obtained. This difference may be partly explained by the varying methodologies of the studies, particularly in terms of sample selection and size. For example, Konofal et al. (2007) found a prevalence estimated at 54% in a sample of 22 subjects whereas Srifuengfung et al. (2020) reported a prevalence of 11% in a sample of 217 subjects [36,37].

The literature also highlights notable variations in the prevalence and clinical profile of RLS among children and adolescents with ADHD across different geographical contexts. For example, the Thai study by Srifuengfung et al. (2020) reported an RLS prevalence of 11% in 217 ADHD patients, with a high proportion of RLS mimics (23%) [36]. This figure is substantially lower than those reported in several Western studies. Several hypotheses may explain these discrepancies: (i) genetic and familial differences (Srifuengfung et al. found a significant association with family history of RLS), (ii) variability in iron deficiency rates across regions, (iii) dietary and environmental factors, (iv) socio-economic influences, and (v) cultural and clinical differences in symptom identification and verbalization, particularly in pediatric populations [36]. Thus, the ADHD–RLS relationship may be influenced not only by shared neurobiological mechanisms but also by regional and cultural determinants. Greater harmonization of diagnostic methods and consideration of these factors are essential to enable accurate international comparisons and to improve screening and management strategies.

Furthermore, recent studies appear to report lower prevalence rates of RLS in children and adolescents with ADHD [36,38,40], which could potentially reflect the impact of the 2013 revision of RLS diagnostic criteria or regional differences in the prevalence of RLS in the study populations [36,38,40]. Indeed, it seems that RLS prevalences in the general population are higher in Europe and North America than in Asia [46]. Finally, ADHD and RLS seem to have a bidirectional relationship (where hyperactivity and impulsivity contribute to sleep disturbances, which in turn worsen daytime inattention and impulsivity) that could help to better understand this high prevalence of RLS in pediatric ADHD subjects highlighted in this systematic review [47].

The link between statistical and epidemiological observations linking these two pathologies and their underlying pathophysiological mechanisms currently remains most advanced in the theory of dopaminergic depletion [48,49,50]. This hypothesis is based on the frequent association of this dopamine deficiency with iron deficiency, iron being fundamental in dopamine metabolism and especially in tyrosine hydroxylation [48,49,50]. The disturbances of these systems remain complex and poorly understood. The fact that dopaminergic agonist therapies seem to improve RLS suggests common pathophysiological links between ADHD and RLS [48,49,50]. Studies focusing on dopaminergic precursors show decreased or normal levels, as well as for dopamine transporters. Regarding receptors, studies have shown decreased activity, especially for D2-type receptors [51,52,53], normal [54], or even increased [55]. It is interesting to note that studies do not cover all types of excitatory and inhibitory dopaminergic receptors. Another study has shown an increase in 3-ortho-methyldopa (the metabolite resulting from the degradation of dopamine by monoamine oxidase B), suggesting a possible hyperdopaminergic mechanism, which contradicts the therapeutic effects mentioned earlier and once again demonstrates the extent of the progress to be made in understanding the involvement of the dopaminergic system in RLS [56], and consequently its link with ADHD.

Despite the lack of sufficient evidence, the cornerstone of pediatric RLS management remains iron supplementation, as per the 2017 guidelines of the International Restless Legs Syndrome Study Group, with ferritinemia targets at >50 ng/mL [57]. It seems that adequate iron stores could optimize the response to psychostimulant treatments for ADHD [58]. As mentioned in the introduction, there is currently no treatment with agreement from European scientific societies or the FDA for the treatment of pediatric RLS [31], and therefore no guidelines for the management of RLS in the context of ADHD [59,60]. Interventional studies on this question are rare. They involve relatively small samples compared to the prevalence of ADHD and RLS and primarily evaluate the effect of dopaminergic agents [59,60]. However, given the potential negative impact of RLS highlighted in this systemic review on the clinical picture of pediatric ADHD subjects, it therefore appears essential to provide combined management of these two pathologies in order to improve the quality of life and daily functioning in this specific subgroup of patients.

### Limitations

This systematic review of the literature has several limitations, starting with the fact that only the PubMed-Medline database was consulted. In addition, it is also important to highlight the limited number of available articles meeting the inclusion criteria for this systematic review and their low scientific quality (Grade C—level 4 according to the French recommendations of the National Agency for Accreditation and Evaluation in Health [integrated with the *Haute Autorité de Santé*] and moderate to severe risk of bias according to the ROBINS-I tool). Furthermore, another major limitation is the lack of distinctions within the samples between children and adolescents, except in the study by Sierra Montoya et al. (2018) [38]. Moreover, there are no studies in the literature specifically and solely focusing on the question of the clinical impact of this comorbidity and whether it would be correlated with therapeutic modifications or not. Thus, to answer these questions, it would be necessary to develop and implement scientific research specifically addressing these issues, especially at the interventional level.

## 5. Conclusions

RLS appears to be a frequent comorbidity in children and adolescents with ADHD, with a prevalence markedly higher than in the general pediatric population. This association may reflect shared pathophysiological mechanisms, particularly involving dopaminergic and iron metabolism. This comorbidity seems to lead to more severe clinical presentations, posing more complex therapeutic challenges for clinicians. Further large-scale studies and interventional trials are needed to clarify this relationship and guide tailored treatment strategies.

## Figures and Tables

**Table 1:** Selected studies.

Studies	Country of Study Study Design Evidence Level	Sample Size	Sample Age	Diagnostic Criteria for ADHD	Diagnostic Criteria for RLS	PSG	Other Relevant Parameters	RLS Prevalence	Main Results	Recommendation Level
Srifuengfung et al. (2020) [36]	Thailand Cross-sectional observational study Level 4	217	3–18 years (average age 9.5 years)	DSM-IV TR or DSM 5	Revised IRLSG criteria (2013)	No	Impact on functioning (6 areas)	11%	23% RLS-mimic’s Binary regression: significant association between the presence of a first-degree biological parent with RLS symptoms and the comorbidity of ADHD and RLS Multivariate regression: effects of RLS on academic functioning and life skills in ADHD subjects	Grade C
Konofal et al. (2007) [37]	France Cross-sectional and retrospective observational study Level 4	22	5–9 years (average age 7.3 years)	DSM-IV	IRLSSG criteria (2003)	No	CPRS, family history of RLS, personal history of iron supplementation, serum ferritin	54%	ADHD symptom severity: trend of higher scores in ADHD+RLS than in ADHD Hyperactivity-impulsivity index: trend towards higher scores in ADHD than in ADHD+RLS Ferritin levels: trend towards lower levels in ADHD+RLS than in ADHD Family history of RLS (58% ADHD+RLS and 10% ADHD) significantly associated with lower ferritin levels and higher ADHD scores History of iron supplementation (50% ADHD+RLS and 20% ADHD) associated with higher ADHD scores	Grade C
Sierra Montoya et al. (2018) [38]	Colombia Cross-sectional observational study Level 4	177	4–18 years (average age 10.25 years)	DSM-IV	IRLSSG criteria (2003)	No	ADHD subtype, other sleep disorders	13.5%	RLS symptoms: 42.7% RLS prevalence stratified by age: 4–8 years (21.8%), 9–13 years (51.3%), and >13 years (25.0%) 93.2% on stimulant medication ADHD subjects with low socioeconomic status had higher risk of RLS	Grade C
Chervin et al. (1997) [39]	United States Cross-sectional observational study Level 4	143 (27 ADHD subjects, 43 others psychiatric disorders, 73 controls)	2–18 years (average age 9 years)	DSM-IV	PSQ	Yes	PSQ, IHS	15%	Snoring: significantly more frequent in ADHD subjects compared to other psychiatric disorders (11%) and controls (9%). Association between snoring severity and ADHD severity RLS prevalence: ADHD (15%), other psychiatric disorders (5%), controls (10%) but differences were not significant No significant differences in RLS severity and sleepiness scores between groups	Grade C
Kapoor et al. (2021) [40]	United States Retrospective study Level 4	66	11.6 ± 3.6 years	DSM 5	ICSD-3	Yes		19.7%	81.1% of parents reported restless sleep in their children 75.8% of children with ADHD had a comorbidity 27.3% of children with ADHD had multiple comorbidities	Grade C
Chervin et al. (2002) [41]	United States Cross-sectional observational study Level 4	866 (98 ADHD)	2–14 years (average age 6.8 years)	DSM-IV	PSQ	No	PSQ, inattention and hyperactivity indices (T-score), CPRS	24%	Hyperactivity index > 60 is associated with higher risk of RLS	Grade C
Silvestri et al. (2009) [42]	Italy Cross-sectional observational study Level 4	55	8.9 ± 2.7 years	DSM-IV	IRLSSG criteria (1995)	Yes	ADHD subtype, CPRS, CTRS, SNAP-IV, structured sleep interview	25.4%	ADHD+RLS compared to ADHD: higher ILRS-RS scores and higher hyperactivity and oppositional scores RLS is more associated with hyperactive ADHD subtype Hyperactive-impulsive ADHD subtype and combined ADHD subtype had more RLS with higher severity than inattentive ADHD subtype	Grade C
Oner et al. (2007) [43]	Türkiye Cross-sectional observational study Level 4	87	6–16 years (average age 9.3 years)	K-SADS-PL (DSM-IV)	IRLSSG criteria (2003)	No	Ferritin levels, behavioral tests (K-SAD-PL, CBCL, TRF, CPRS and CTRS)	33.3%	No difference between boys and girls Comparison of ADHD with/without RLS: no difference in behavioral tests Iron deficiency significantly more common in ADHD+RLS	Grade C
Silvestri et al. (2007) [44]	Italy Cross-sectional observational study Level 4	42	8.9 ± 2.8 years	DSM-IV	IRLSSG criteria (2003)	Yes	ADHD subtype, ADHD-RS, CTRS, CPRS, SNAP-IV, structured sleep interview	26.0%	Significant correlation between RLS diagnosis and inattention, impulsivity/hyperactivity, oppositional behaviors scoring	Grade C

ADHD = Attention Deficit Hyperactivity Disorder, RLS = Restless Legs Syndrome, PSG = Polysomnography, K-SADS-PL = Kiddie-SADS-Present and Lifetime, PSQ = Pediatric Sleep Questionnaire, ICSD-3 = International Classification of Sleep Disorders-Third Edition, IRLSSG = International Restless Legs Syndrome Study Group, HIS = inattention/hyperactivity score, ADHD-RS = ADHD Rating Scale, CBCL = Child Behavior Checklist, TRF = Teacher’s Report Form, CPRS = Conners Parent Rating Scale, CTRS = Conners Teacher Rating Scale, SNAP-IV = Swanson, Nolan and Pelham Rating Scale-IV, ILRS-RS = International Restless Legs Syndrome-Rating Scale.

**Table 2:** Potential biases of selected studies.

Studies	D1	D2	D3	D4	D5	D6	D7	Global Risk
Srifuengfung et al. (2020) [36]	Moderate	Low	Low	Not applicable	Low	Moderate	Low	Moderate
Konofal et al. (2007) [37]	Severe	Moderate	Moderate	Not applicable	Low	Moderate	Moderate	Severe
Sierra Montoya et al. (2018) [38]	Severe	Moderate	Moderate	Not applicable	Low	Moderate	Moderate	Severe
Chervin et al. (1997) [39]	Severe	Moderate	Moderate	Not applicable	Low	Moderate	Moderate	Severe
Kapoor et al. (2021) [40]	Moderate	Moderate	Low	Not applicable	Low	Moderate	Low	Moderate
Chervin et al. (2002) [41]	Moderate	Low	Moderate	Not applicable	Moderate	Moderate	Low	Moderate
Silvestri et al. (2009) [42]	Moderate	Moderate	Low	Not applicable	Low	Moderate	Low	Moderate
Oner et al. (2007) [43]	Moderate	Moderate	Moderate	Not applicable	Low	Moderate	Low	Moderate
Silvestri et al. (2007) [44]	Moderate	Moderate	Low	Not applicable	Low	Moderate	Low	Moderate

D1: bias due to confounding; D2: bias due to selection of participants; D3: bias in classification of interventions; D4: bias due to deviations from intended interventions; D5: bias due to missing data; D6: bias in measurement of outcomes; D7: bias in selection of the reported results.

## Data Availability

The original contributions presented in this study are included in the article. Further inquiries can be directed to the corresponding author.
